# A case report of dipeptidyl peptidase 4 inhibitor-related kidney disease combined with renal cancer

**DOI:** 10.3389/fneph.2024.1409098

**Published:** 2024-07-29

**Authors:** Shigekazu Kurihara, Naoki Sawa, Keiichi Sumida, Daisuke Ikuma, Yuki Oba, Hiroki Mizuno, Akinari Sekine, Masayuki Yamanouchi, Eiko Hasegawa, Tatsuya Suwabe, Shinji Urakami, Kei Kono, Keiichi Kinowaki, Kenichi Ohashi, Yutaka Yamaguchi, Yoshifumi Ubara

**Affiliations:** ^1^ Nephrology Center and the Okinaka Memorial Institute for Medical Research, Toranomon Hospital, Tokyo, Japan; ^2^ Department of Urology, Toranomon Hospital, Tokyo, Japan; ^3^ Department of Pathology, Toranomon Hospital, Tokyo, Japan; ^4^ Department of Human Pathology, Tokyo Medical Dental University, Tokyo, Japan; ^5^ Yamaguchi’s Pathology Laboratory, Chiba, Japan

**Keywords:** kidney biopsy, renal cell carcinoma, dipeptidyl peptidase (DPP) 4 inhibitors, thrombotic microangiopathy (TMA)-like lesion, end-stage renal failure

## Abstract

A kidney biopsy was performed in a 64-year-old woman with type 2 diabetes mellitus and less than 1 g of proteinuria who rapidly progressed to end-stage renal failure after approximately 2 years of treatment with two dipeptidyl peptidase 4 (DPP-4) inhibitors for type 2 diabetes mellitus. The biopsy revealed not only a coincidental diagnosis of renal cell carcinoma, which was not evident on pre-biopsy computed tomography, but also severe thrombotic microangiopathy (TMA)-like glomerular endothelial cell damage in the noncancerous areas. These results suggest that DPP4 inhibitors may have been involved in two kidney diseases.

## Introduction

Kidney biopsies were performed to investigate the cause of proteinuria, hematuria, and renal dysfunction. However, they are usually only conducted after diagnostic imaging tests, such as ultrasound and computed tomography (CT) have confirmed the absence of morphological abnormalities, such as renal tumors. Consequently, renal tumors are usually not diagnosed by biopsy. The Japanese Guidebook on Kidney Biopsy, which was developed from the results of a survey on the indications for kidney biopsy, describes the use of biopsy for diagnosing renal tumors. Some reports describe the incidental diagnosis of neoplasms, including intravascular lymphoma, by kidney biopsy; however, reports of incidental diagnosis of renal cancer are scarce (1–3).

Here, we report a case in which renal cancer was incidentally diagnosed by kidney biopsy performed to investigate the pathogenesis of rapid decline in renal function in a patient with type 2 diabetes mellitus (T2D).

## Patient information, clinical findings and timeline

A 64-year-old woman with progressive renal impairment was admitted to our hospital. She was diagnosed with T2D at the age of 45 years and maintained good control of blood glucose with diet therapy only. A right adrenal tumor was found at the age of 46 years and was diagnosed as nonfunctional.

**Figure 1:**
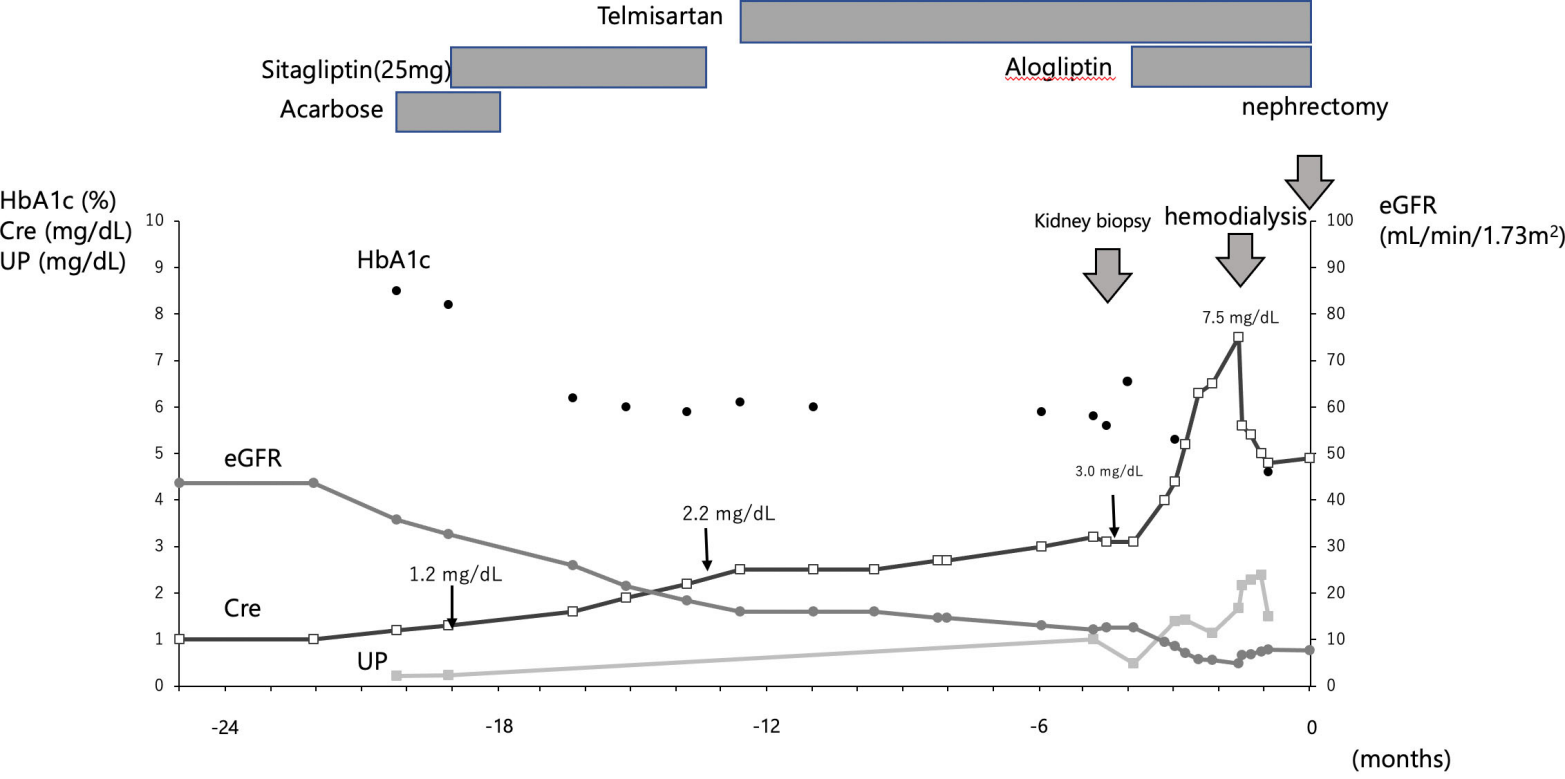
Clinical course. The figure shows the clinical course up to nephrectomy. The timing of kidney biopsy, hemodialysis and nephrectomy is indicated by thick arrows. Sitagliptin was given at Cre 1.2 mg/dL and was stopped when Cre fell to 2.2 mg/dL; alogliptin was started at Cre 3.0 mg/dL; hemodialysis was started when Cre reached 7.5 mg/dL.

Fourteen months before the current admission, the laboratory findings were as follows: serum creatinine (Cre), 1.2 mg/dL; hemoglobin A1c (HbA1c), 8.5%; urinary protein, 0.3 g/gCre; and erythrocytes in the urinary sediment, more than 30 per high power field. Sitagliptin, a dipeptidyl peptidase 4 (DPP4) inhibitor, was administered at a dose of 25 mg to treat hyperglycemia, and the HbA1c level subsequently decreased to 6.1%. Eight months later, sitagliptin was discontinued because renal function had declined, as shown by a Cre value of 2.2 mg/dL. The subsequent progression of renal function decline was slow (**Figure 1**).This patient was admitted to investigate the cause of kidney impairment.

On admission, the patient was 147 cm tall and weighed 45.7 kg. Her blood pressure was 155/66 mmHg, heart rate was 82 beats/min, and body temperature was 36.4°C. The heart and breath sounds were normal; however, edema was present in the lower extremities. Funduscopic examinations revealed no retinopathy.

The results of blood chemistry tests were as follows: serum albumin, 3.5 g/dL; serum creatinine, 3.0 mg/dL, estimated glomerular filtration rate, 17.6 ml/min/1.73 m^2^; C-reactive protein, less than 0.1 mg/dL; immunoglobulin G (IgG), 1105 mg/dL; IgA, 301 mg/dL; IgM, 54.0 mg/dL; total complement activity (assessed as CH50), 57 U/mL; antinuclear antibody, negative; fasting plasma glucose, 97 mg/dL; HbA1c, 6.0%; glycoalbumin, 16.8%; total cholesterol, 170 mg/dL; and triglycerides, 121 mg/dL. The urinary protein excretion was 0.84 g/day, and the urinary sediment contained more than 90 erythrocytes per high-power field. Ultrasonic examination revealed that the maximum diameter of both kidneys was 10 cm, no atrophy was observed, and no renal tumor was detected. The right kidney was then biopsied.

## Diagnostic assessment (kidney biopsy)

**Figure 2:**
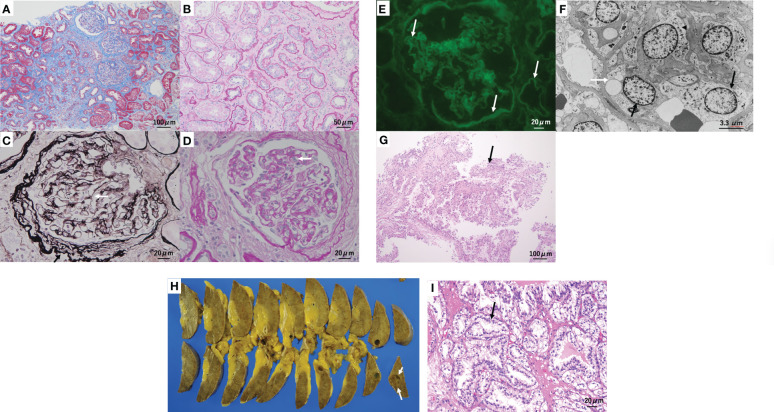
Kidney biopsy and surgical specimen. **(A, B)** Tubulointerstitial fibrosis and tubular atrophy are observed in approximately 70% of the cortical area. **(A)** Masson trichrome staining. **(B)** Periodic acid–Schiff (PAS) staining. **(C)** Many of the preserved glomeruli show prominent fibrotic thickening (black arrow) of the glomerular basement membrane (GBM) of Bowman’s capsule and duplication (white arrow) of the GBM. Periodic acid methenamine silver and Masson staining. **(D)** Glomerular endothelial cell proliferation (arrow) was observed. PAS staining. **(E)** Immunofluorescence microscopy reveals linear fluorescence of immunoglobulin G (arrow) along the GBM, Bowman’s capsule, and tubular basement membrane. **(F)** Electron microscopy reveals endothelial cell proliferation (black arrow) with mesangiolysis (arrowhead) and subendothelial edema (white arrow). The GBM thickened to a width of 400–500 nm. **(G)** Two biopsy specimens showed clear cell renal cell carcinoma(arrow). Hematoxylin and eosin staining: original magnification ×200. **(H)** split section of the surgical specimen. A tumor(arrow), 9–10 mm in size, was found in the surgical specimen in the lower pole of the right kidney. **(I)** Histology also confirmed clear cell renal cell carcinoma(arrow). Hematoxylin and eosin staining; original magnification ×400.

Five specimens were obtained by kidney biopsy. Light microscopic examination of the three specimens revealed global sclerosis in four out of 23 glomeruli. Tubulointerstitial fibrosis and atrophy with thickening of the tubular basement membrane were observed in approximately 70% of the cortical area (**Figures 2A, B**). Many of the preserved glomeruli showed fibrotic thickening of the basement membrane of the Bowman’s capsule (**Figure 2C**). The mesangium matrix was obscure. Duplication of the glomerular basement membrane (GBM) (**Figure 2C**) and endothelial cell proliferation were observed (**Figure 2D**). Arteriolar hyalinosis was mild, and fibroelastosis of the interlobular arteries was mild-to-moderate. Immunofluorescence microscopy revealed linear fluorescence of IgG along the glomerular basement membrane (GBM), Bowman’s capsule, and tubular basement membrane (**Figure 2E**), but staining for IgA, IgM, C3, and C1q was negative. Electron microscopy revealed endothelial cell proliferation with mesangiolysis and subendothelial edema. The GBM was thickened to a width of 400–500 nm (**Figure 2F**); however, electron-dense deposits were not observed. According to Tervaert’s pathologic classification of diabetic nephropathy(DN) (4), the glomerular classification was Class IIa (with mild mesangial expansion),interstitial lesions IFTA score 3(>50%), interstitial inflammation score 1, vascular lesions arteriolar hyalinosis score 1, and arteriosclerosis score 1. In this case, the glomerular lesions were mild, but the interstitial lesions were moderate to severe. The glomerular lesions in this case were characterized by a high degree of thrombotic microangiopathy (TMA)-like endothelial cell damage, consistent with glomerular microangiopathy. Although the glomerular lesions associated with diabetic nephropathy were mild, advanced interstitial lesions and TMA-like glomerular microangiopathy may have been related to the renal prognosis in this case. This TMA-like lesion is not usually observed in diabetic nephropathy. This is similar to drug-induced nephropathy caused by recent anticancer drugs (5), which will be discussed later.

Two biopsy specimens showed clear cell renal cell carcinoma (RCC), characterized by proximal tubular cell-like tumor cells with pale or acidophilic cytoplasms (**Figure 2G**). The border between the cancerous and non-cancerous areas was clearly defined by a fibrous capsule.

## Clinical finding

**Figure 3:**
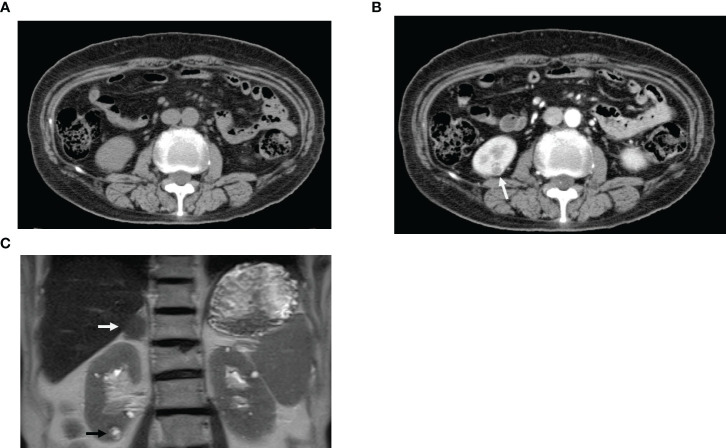
Diagnostic imaging. **(A)** Computed tomography (CT) scan before contrast enhancement. **(B)** CT scan immediately after contrast enhancement; white arrow indicates hypervascular staining (Equivalent to renal cancer). **(C)** Magnetic resonance imaging; Black arrow indicates mass, 9 mm in size (Equivalent to renal cancer) in the lower pole of the right kidney. White arrow confirmed the presence of a right adrenal tumor.

After kidney biopsy, the spread of the tumor was re-examined using contrast-enhanced CT. A hypervascular stain, 8 mm in size, was observed in the lower pole of the right kidney (**Figure 3**). Magnetic resonance imaging (MRI) T2-weighted imaging showed a mass, 9 mm in size, that had a higher intensity than the renal parenchyma and was located in the lower pole of the right kidney. MRI also confirmed the presence of a right adrenal tumor, which was still the same size even after 20 years. Dehydroepiandrosterone sulfate, adrenocorticotropic hormone, aldosterone, adrenaline, noradrenaline, dopamine, and renin were all within their respective reference ranges.

## Therapeutic intervention and follow-up and outcomes

After kidney biopsy, HbA1c began to rise again and another DPP-4 inhibitor, alogliptin, was administered. Alogliptin, a DPP4 inhibitor, was started, and the decline in renal function progressed to Cre 7.5 mg/dL; hemodialysis was initiated for end-stage renal failure. Two months later, the patient underwent a radical laparoscopic right nephrectomy and adenectomy. No drugs were used that might contribute to TMA-like lesions, except for DPP4 inhibitors (**Figure 1**).

The surgical specimen contained a tumor, 9 mm in size, in the lower pole of the right kidney (**Figure 2H**). Histological examination revealed clear cell RCC. The size of the tumor cell nucleus was equivalent to that of the nucleus of the surrounding tubules (grade 1, Fuhrman grade 1, INFα, pT1a) (**Figure 2I**). TNM classification was cT1aN0M0. The non-cancerous tissue of the resected specimen consisted of highly fibrotic interstitial lesions and sclerotic glomeruli corresponding to end-stage renal failure. The pathophysiology of the development of renal failure in a rather short period of time is noteworthy. No invasion of the surrounding veins or lymph vessels was observed. The adrenal gland showed adrenocortical adenoma.

## Discussion

This article contains two elements: First, the RCC revealed by kidney biopsy, and second, the rapid progression of renal dysfunction in the context of noncancerous renal tissue.

This was the first case of incidental diagnosis of RCC on kidney biopsy at our hospital; however, from 1985 to April 2022, we performed 7497 kidney biopsies for pathological diagnosis of kidney disease, including allogeneic transplants (this number does not include biopsies of renal tumors identified by diagnostic imaging before biopsy).

We searched for reports of similar cases in which a malignancy was incidentally diagnosed on kidney biopsy. Pankhurst et al. reported that among 11880 kidney biopsies, including allogeneic transplants, performed from 1982 through 2003, 25 identified accidental neoplasms. Of the 25 cases, two were clear cell RCC, one was *in situ* carcinoma in a collecting duct, and the remaining 22 were papillary neoplasms (6). Sperati et al. reported a case of papillary RCC (Fuhrman grade 3) that was incidentally diagnosed by percutaneous biopsy. The diameter of papillary RCC was 1.1 cm. At their institution, 9330 kidney biopsies were performed between 1984 and 2008, and a total of three cases (0.03%) had evidence of possible malignancy with unremarkable kidney imaging (results of kidney biopsy of transplanted kidneys were excluded). The other two cases involved a 51-year-old man with tubulopapillary hyperplasia and a 47-year-old woman with chromophobe RCC associated with fibrillary glomerulonephritis (7). Jamis-Dow CA examined the limits of detection of renal cancer on CT and ultrasound scans by analyzing a database of 21 patients with von Hippel-Lindau disease or hereditary papillary renal cancer. Contrast-enhanced CT and ultrasound detection rates were 47% and 0% for lesions of 0–5 mm, respectively, and 60% and 21% for lesions of 5–10 mm (8). O’Connor et al. reported that renal cancers < 3 cm are often difficult to diagnose using unenhanced CT (9).

Looking back at the clinical course of this case, it is clear that glycemic control improved after sitagliptin administration, but renal function progressively deteriorated. After discontinuation of sitagliptin, the progression of renal function loss slowed, but the patient rapidly progressed to end-stage renal failure following the administration of alogliptin. This suggests that DPP4 inhibitors may contribute to the progression of renal failure. To answer this question, we began with a detailed review of kidney biopsy results.

Regarding the causes of renal dysfunction, we searched for articles discussing the association between the general prognosis of diabetic nephropathy and renal histology findings. We found that Mise et al. reported a better 10-year prognosis for class IIa in Tervaert’s pathological classification of diabetic nephropathy than for classes IIb, III, and IV (10). If a diagnosis of class IIa of Tervaert’s pathological classification is made, the decline in renal function progresses slowly. However, renal function declined in the present case; therefore, we considered the possible causes. Kidney biopsy showed fewer glomeruli with total nodular sclerosis and more damage to glomeruli that were spared from sclerosis compared to the progression of loss of renal function. Therefore, we focused on the presence of TMA-like lesions in the preserved glomeruli. Eremina et al. reported that patients treated with bevacizumab, a humanized monoclonal antibody against vascular endothelial growth factor (VEGF)A, had TMA-like glomerular lesions characterized by glomerular endothelial cell damage. They explained that VEGF released from podocytes protects endothelial cells, but the drug suppresses VEGF, resulting in endothelial cell damage. They also reported that the drug also induces hypertension, which worsens the glomerular lesions. The glomerular lesions described by Eremina et al. are similar to those described by us in the present study and may therefore represent a common type of drug-induced glomerular lesion, although the drugs used are different and the mechanisms are different (5); therefore, we reviewed our case in the context of drug-induced nephropathy. The clinical course of the patient showed progressive deterioration of renal function after the use of the two DPP-4 inhibitors. Suenaga et al. recently reported glomerular lesions of TMA-like glomerular microangiopathy as well as bullous pemphigoid after DPP4 inhibitor treatment in type 2 diabetic patients (11, 12). In terms of the mechanism, the following article does not discuss direct glomerular damage, but may be of interest as endothelial regeneration of blood vessels may lead to glomerular endothelial damage. Brenner et al. reported that sitagliptin, a DPP-4 inhibitor, prevents the degradation of the chemokine SDF-1α and improves the recruitment of regenerative circulating CXCR4+ progenitor cells that mediate local endothelial cell proliferation without adversely affecting the structure of the vessel wall, using a mouse carotid artery injury model, and it can promote endothelial regeneration after acute endothelial injury (13). DPP-4 inhibitors may induce endothelial cell proliferation in the glomerulus, leading to TMA-like lesions.

DPP-4 inhibitors are effective in controlling blood glucose levels, have a low risk of hypoglycemia, are well tolerated in patients with T2D and diabetic kidney disease (DKD), and are expected to reduce the progression of diabetic nephropathy. Some studies reported significant reductions in albuminuria or less progression of albuminuria, while others reported no significant efficacy in inhibiting renal progression of DKD. We were unable to find any publications on the long-term renal prognosis associated with DPP4 inhibitors (14, 15). Although the glomerular lesions in this case were mild according to the diabetic nephropathy criteria based on mesangial matrix expansion, TMA-like lesions in the glomeruli and severe interstitial lesions, which were not included in the diabetic nephropathy criteria, were clearly involved in the renal damage in this case, suggesting that DPP4 inhibitors were involved in renal damage via a mechanism different from that of the conventional mechanism.

It is possible that the DPP4 inhibitor may have contributed to the progressive decline in renal function in this case despite good glycemic control during DPP4 inhibitor treatment. In our case, renal cancer was found incidentally after sitagliptin treatment. The relationship between this drug and the development of renal cancer is not yet clear as this is the only case of this drug. However, the following article on DPP4 inhibitors and malignant tumors is considered an interesting pathogenesis. Yang et al. studied human and mouse breast cancer cell lines and DPP4 inhibitor-treated mouse allograft models. They reported that DPP-4 inhibitors may promote cancer progression via induction of the CXCL12/CXCR4/mTOR axis, which is also important for vascular damage in cancer tissue. They did not discuss renal lesions, but DPP4 inhibitors and renal cancer may be considered in the future (16). Akashi et al. suggested that DPP4 inhibitors may improve vascular endothelial function and heal impaired vessels by increasing the number of circulating endothelial progenitor cells (EPCs). In this case, the mild arteriohyalinosis in spite of the history of DM may have contributed to the prevention of small renal arteries by DPP4 inhibitors, whereas in the glomeruli, it was speculated that the increase in the number of endothelial cells may have caused TMA-like lesions (17).

In conclusion, we encountered a case of renal cancer less than 1 cm in size that was incidentally diagnosed by kidney biopsy. Nephrologists usually perform kidney biopsy to investigate the cause of proteinuria, hematuria, or renal function loss and are not aware of malignant tumors. It is important to consider the possibility of microscopic renal cancer, although this is less common. The noncancerous renal area is a TMA-like lesion associated with DPP4 inhibitor administration, which may contribute to the rapid decline in renal function. The patient already had hematuria before starting sitagliptin. This could suggest that renal cancer may have been an inducer of TMA-like lesions under DPP4-inhibitor treatment. We should not conclude from this single case report that DPP4-inhibitors are associated with TMA-like lesions or renal cancer. However, awareness of these conditions may be helpful in interpreting similar cases.

## Data availability statement

The raw data supporting the conclusions of this article will be made available by the authors, without undue reservation.

## Ethics statement

The studies involving humans were approved by Toranomon Hospital institutional review board. The studies were conducted in accordance with the local legislation and institutional requirements. Written informed consent for participation in this study was provided by the participants’ legal guardians/next of kin. Written informed consent was obtained from the individual(s) for the publication of any potentially identifiable images or data included in this article.

## Author contributions

YU: Conceptualization, Data curation, Formal analysis, Funding acquisition, Investigation, Methodology, Project administration, Resources, Software, Supervision, Validation, Visualization, Writing – original draft, Writing – review & editing. SK: Conceptualization, Writing – original draft. NS: Conceptualization, Writing – review & editing. KS: Investigation, Writing – review & editing. DI: Data curation, Writing – review & editing. YO: Conceptualization, Writing – review & editing. HM: Validation, Writing – review & editing. AS: Formal analysis, Writing – review & editing. MY: Data curation, Writing – review & editing. EH: Data curation, Writing – review & editing. TS: Data curation, Writing – review & editing. SU: Supervision, Writing – review & editing. KKo: Data curation, Writing – review & editing. KKi: Data curation, Writing – review & editing. KO: Supervision, Writing – review & editing. YY: Supervision, Writing – review & editing.
